# RHO GTPase family in hepatocellular carcinoma

**DOI:** 10.1186/s40164-022-00344-4

**Published:** 2022-11-08

**Authors:** Tiantian Wang, Dean Rao, Chengpeng Yu, Jiaqi Sheng, Yiming Luo, Limin Xia, Wenjie Huang

**Affiliations:** 1 Hubei Key Laboratory of Hepato-Pancreato-Biliary Diseases, Hepatic Surgery Center, Tongji Hospital, Tongji Medical College, Clinical Medicine Research Center for Hepatic Surgery of Hubei Province, Key Laboratory of Organ Transplantation, Ministry of Education and Ministry of Public Health, Huazhong University of Science and Technology, Wuhan, 430030 Hubei China; 2 Department of Gastroenterology, Institute of Liver and Gastrointestinal Diseases, Hubei Key Laboratory of Hepato-Pancreato-Biliary Diseases, Tongji Hospital of Tongji Medical College, Huazhong University of Science and Technology, Wuhan, 430030 Hubei China

**Keywords:** RHO GTPases, RHOA, RAC1, CDC42, Cancer, Hepatocellular carcinoma

## Abstract

RHO GTPases are a subfamily of the RAS superfamily of proteins, which are highly conserved in eukaryotic species and have important biological functions, including actin cytoskeleton reorganization, cell proliferation, cell polarity, and vesicular transport. Recent studies indicate that RHO GTPases participate in the proliferation, migration, invasion and metastasis of cancer, playing an essential role in the tumorigenesis and progression of hepatocellular carcinoma (HCC). This review first introduces the classification, structure, regulators and functions of RHO GTPases, then dissects its role in HCC, especially in migration and metastasis. Finally, we summarize inhibitors targeting RHO GTPases and highlight the issues that should be addressed to improve the potency of these inhibitors.

## Introduction

The RHO GTPases form a subfamily of the RAS superfamily of GTP-binding proteins with a size of 21 to 25 kDa which are found in all eukaryotic cells. The first RHO GTPase protein was discovered in the abdominal ganglia of Aplysia in 1985 [1]. RHO GTPases have a conserved primary structure with 50–55% sequence similarity to each other [2]. However, it was observations reported years later that put forward the perception of the cellular function of RHO GTPases, mainly linked to the actin cytoskeleton [3–5], and further studies demonstrated that they regulate many other signal transduction pathways. They are involved in the modulation of cell proliferation control, cell polarity, development, vesicular transport pathways and other aspects of cell biology [6]. Conversely, the dysregulation of RHO GTPases is associated with various diseases such as inactivating mutation in T cell lymphoma [7], overexpression in hypertension [8], and abnormal activation in arthritis [9].

Hepatocellular carcinoma (HCC) is the leading cause of cancer-related death worldwide, especially the overall survival of patients with advanced HCC should be improved [10]. Exploring the pathogenesis of HCC may help us out of this woods. Strikingly, aberrant expression of RHO GTPase was found to contribute to HCC progression [11], especially migration and metastasis. Furthermore, RHO GTPases are regarded as potential diagnostic biomarkers or therapeutic targets for cancer [12, 13]. However, the underlying mechanisms through which RHO GTPases contribute to HCC initiation and progression remain poorly understood.

In this review, we first introduce the classification, structure, regulators and functions of RHO GTPases, then dissect their role in HCC initiation and development, especially in migration. Finally, we discuss inhibitors targeting the RHO GTPases and highlight the issues that should be addressed to improve the potency of these inhibitors.

## RHO GTPase family in biology

According to their structural similarity, sequence, and intracellular functions, the RAS superfamily of GTPases is divided into five main groups: RAS, RHO, RAB, ARF and RAN. Among them, RHO family GTPases have recently been expanded to over 20 members [14], and some of them have been extensively studied, including RHOA, RAC1 and CDC42. What’s more, most of them are highly conserved among eukaryotic species [15]. Activated by extracellular factors such as soluble molecules, adhesion interactions, and mechanical stress, RHO GTPases can initiate signaling cascades that span a wide range of targets or effectors, including kinases and scaffold/adaptor-like proteins [16]. The activation and inactivation of RHO GTPases is mainly regulated by three upstream factors: the guanine nucleotide dissociation inhibitors (GDIs), guanine nucleotide exchange factors (GEFs), and GTPase-activating proteins (GAPs).

### RHO Family GTPases

**Fig. 1:**
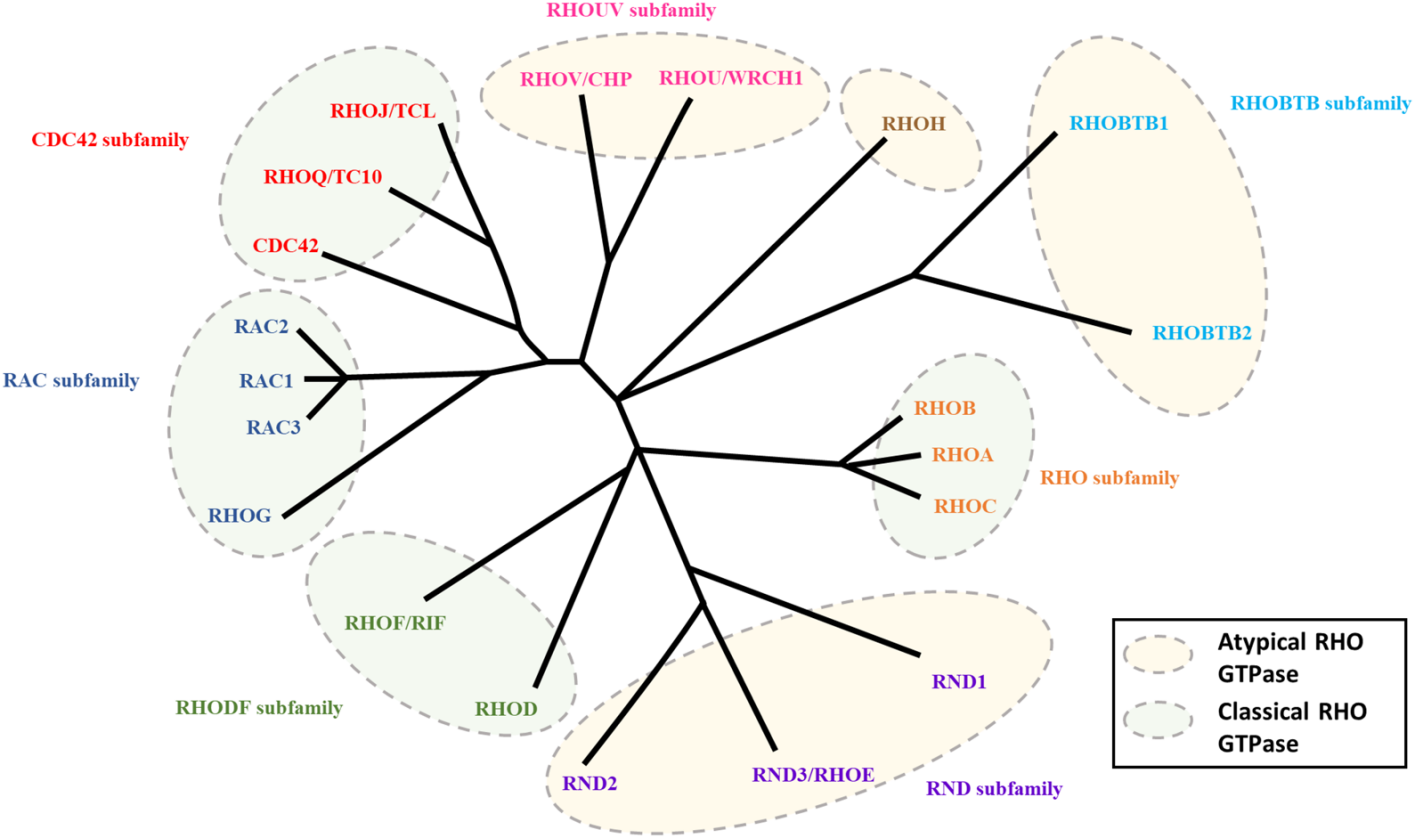
RHO GTPase family. The unrooted phylogenetic tree of the RHO GTPase family was based on the Clustal Omega program alignment of the amino-acid sequences of the 20 RHO GTPase proteins. RHO GTPases can be classified into eight subfamilies: RHO, RAC, CDC42, RHODF, RHOUV, RND, RHOH and RHOBTB. These subfamilies are highlighted with circles and labeled on the right side. The classical RHO GTPases include four subfamilies: RHO, RAC, CDC42 and RHODF, which are regulated by the GDP/GTP cycle. The atypical RHO GTPases comprise the RHOUV, RND, RHOH and RHOBTB subfamilies. These GTPases are regulated by gene expression, localization, phosphorylation, and/or protein stability and not by GEF and GAP

**Fig. 2:**
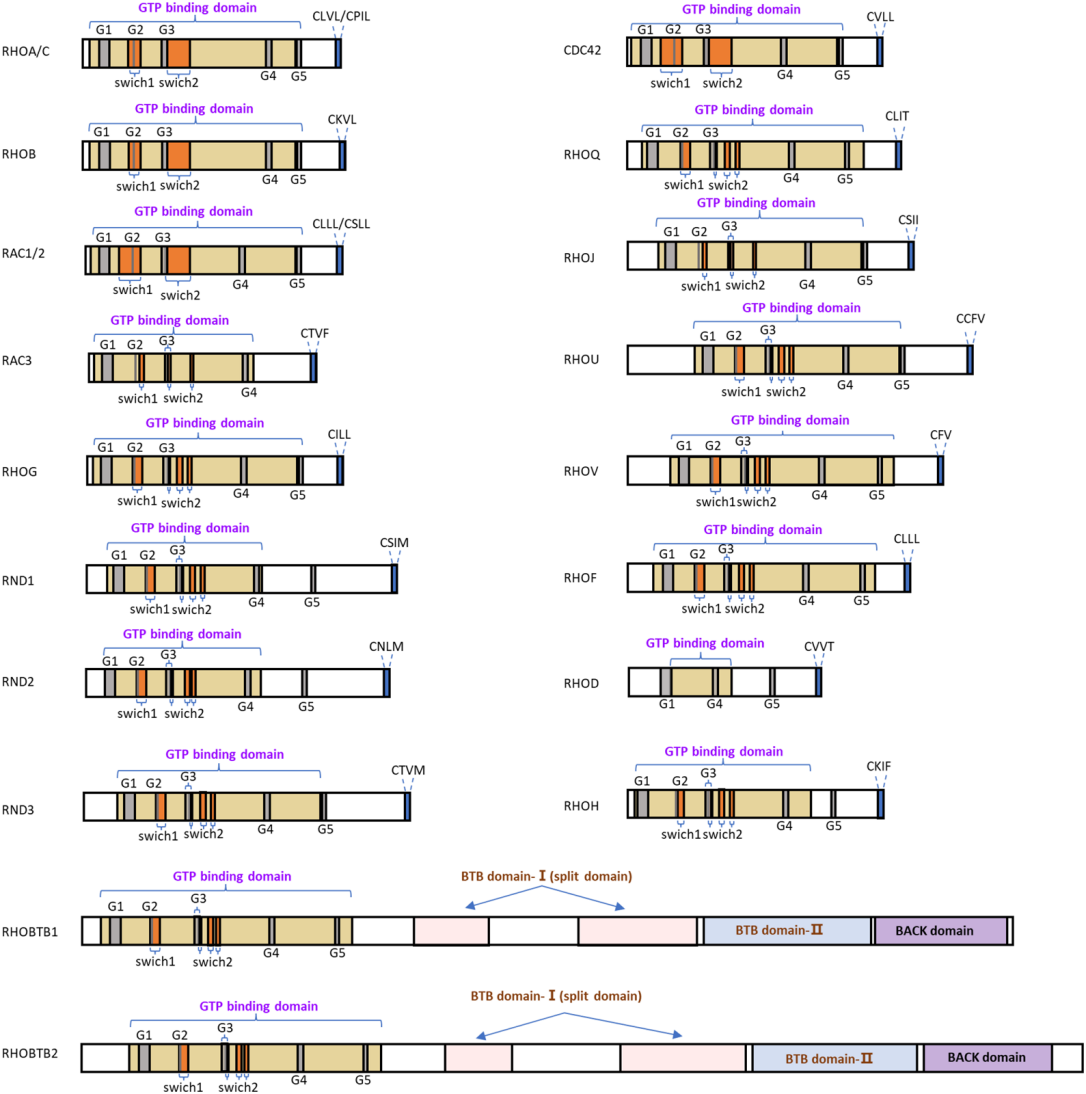
Domains of RHO GTPases. RHO GTPases typically contain a G domain, which is a conserved GDP/GTP-binding domain that participates in nucleotide binding and hydrolysis. Switch I/II regions of RHO GTPase are the common binding sites for GEFs, GDIs, GAPs, or effectors, and undergo conformational changes during the nucleotide exchange and hydrolysis cycle. The C-terminal hypervariable region ends with a common sequence, which is known as CAAX, and modification at this sequence is crucial for subcellular localization of RHO GTPases

RHO GTPases can be classified into eight subfamilies based on their sequence homology, including RHO (RHOA–RHOC), RAC (RAC1–RAC3 and RHOG), CDC42 (CDC42, RHOJ/TCL and RHOQ/TC10), RHODF (RHOD and RHOF/RIF), RHOUV (RHOU/WRCH1 and RHOV/CHP), RND (RND1, RND2, RND3/RHOE), RHOH, and RHOBTB (RHOBTB1 and RHOBTB2) (Fig. 1). RHO GTPases typically contain a G domain and a C-terminal hypervariable region (Fig. 2). The G domain consists of five conserved sequence motifs, G1 to G5, which constitute a conserved GDP/GTP-binding domain that participates in nucleotide binding and hydrolysis. The C-terminal hypervariable region ends with a common sequence, which is known as CAAX (C: cysteine, A: any aliphatic amino acid, and X: any amino acid) [17] and modification at this sequence are crucial for the subcellular localization of RHO GTPases [18]. Switch I/II regions of RHO GTPase are the common binding sites for GEFs, GDIs, GAPs, or effectors, and change their conformation during the nucleotide exchange and hydrolysis cycle (Fig. 2) [17]. RHO GTPases are generally expressed ubiquitously, but there are exceptions such as RHOH, RAC2, RHOBTB and RND3 which express in the specific cell [15].

RHO GTPases can also be classified into a classical subfamily and an atypical subfamily bases on their structure, kinetic properties and other features. The former includes the four subfamilies RHO, RAC, CDC42 and RHODF, which are regulated by GDP/GTP exchange and exert the main functions of the RHO GTPase family [19]. By contrast, some RHO GTPase members do not follow this classical GTPase cycle, and are consequently considered atypical. Some of them have a remarkably enhanced intrinsic GDP/GTP activity, while some fail to hydrolyze GTP, and were respectively named the fast-cycling RHO GTPases (RHOUV), and hydrolysis-deficient RHO GTPases (RND, RHOH and RHOBTB) [20]. In addition to control by GEFs and GAPs, these GTPases are regulated at the level of gene expression, localization, phosphorylation, and/or protein stability, and their functions involve additional domains that are not present in typical RHO proteins [21].

### Upstream regulators of RHO GTPases

**Fig. 3:**
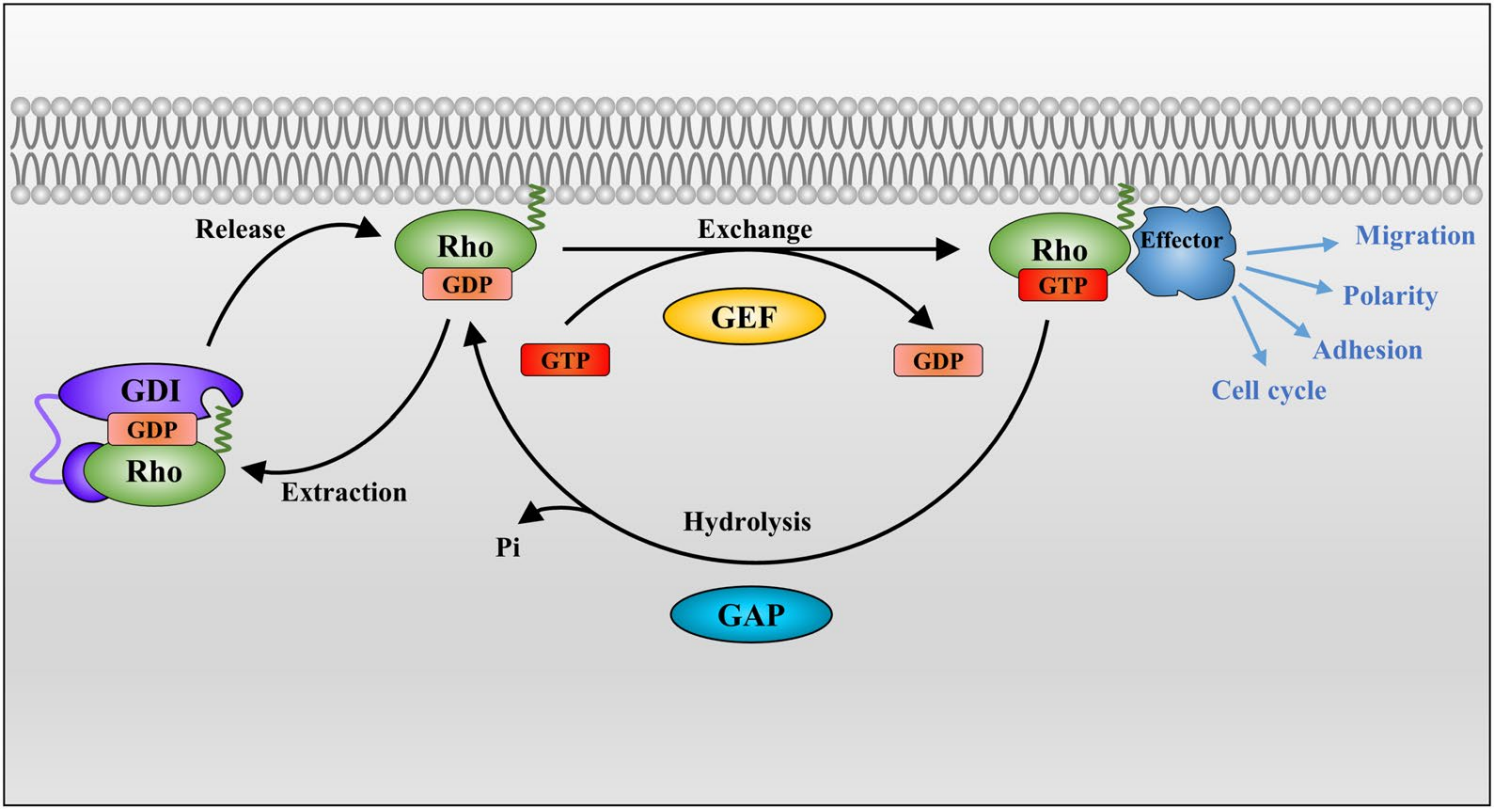
The classical RHO GTPase cycle. RHOGEFs bind to the GDP-bound form RHO GTPase and induce the exchange of GDP for GTP to activate RHO. Subsequently, the conformation of RHO GTPases changes, which allows them to interact with their specific effectors and triggers biological effects such as cell migration, polarity and adhesion. RHOGAPs bind to the GTP-bound form of RHO GTPases to promote their intrinsic GTP hydrolysis activity, thereby acting as an inhibitor of RHO GTPases. RHOGDIs can extract geranylgeranylated RHO GTPases from the membrane and inhibit nucleotide exchange and hydrolysis to restrict GDP/GTP cycling. RHOGDIs maintain inactive pools of RHO GTPases in the cytosol and protect the prenyl group of the RHO GTPase in the hydrophobic pocket, preventing RHO GTPases from inappropriate activation, misfolding, or degradation

Similar to other members of the RAS superfamily, the activity of RHO GTPases is determined by the ratio of their GTP/GDP-bound forms inside the cell. Although the RHO switch itself that transmits extracellular cues to intracellular signaling pathways is straightforward, it is intricately regulated by three main classes regulatory proteins (Fig. 3): GEFs, GDIs, and GAPs. Among them, GEFs positively regulate RHO GTPases, while GAPs and GDIs exert negative regulation. In humans, there are 85 GEFs, 66 GAPs, and 3 GDIs, which mediate the precise regulation of RHO GTPases [17].

#### GEFs

RHO GTPases are generally activated by GEFs, which can bind to the GDP-bound form, destabilize the GDP-GTPase complex and stabilize a nucleotide-free reaction intermediate by deforming the phosphate-binding site of RHO GTPase at the same time, instead of directly stimulating the binding of the RHO GTPase to GDP/GTP [22]. Then, the high intracellular concentration of the nucleotide becomes the only determinant of the binding of RHO GTPase to GTP. As the free nucleotide-binding site of RHO GTPase has a similar affinity for GTP and GDP, while the GTP concentration is tenfold higher than that of GDP, RHO GTPase will bind to GTP and be activated [22]. Subsequently, the bound GTP replaces the GEF and the conformation of RHO GTPase changes again. This process enables RHO GTPases to interact with their specific effectors, thereby facilitating downstream signal transduction.

According to their structure, GEFs are classified into two families, the dedicator of cytokinesis (DOCK) homology region domain family and the DBL-homology (DH) domain family [23]. The majority of RHO GEFs belong to the DBL family, while the DOCK family functions as GEFs for CDC42 and/or RAC, but not RHOA [24]. In addition, it is believed that the action of GEFs is specific not only to their substrates, i.e. RHO GTPases, but also to the cell types and upstream signals [25]. For example, GEFs act immediately upstream of RHO GTPases, providing a direct link between the activation of RHO and various cell surface receptors for growth factors, adhesion molecules, cytokines, and G protein-coupled receptors [23].In addition, RHO GEFs play an essential scaffolding function and coordinate downstream signaling in response to upstream cell stimuli by interacting with a definite set of targets and bringing these effector proteins into the vicinity of the RHO GTPases which they activate [24].

There are four times as many RHO GEFs as RHO GTPases, allowing a more rigorous spatiotemporal control of various activities. Several GEFs have high specificity for a single GTPase (such as FGD1, which activates CDC42), while others may not be as specific (such as α-PIX, which activates both CDC42 and RAC) [23]. However, it remains challenging to predict the substrate specificity of most GEFs.

#### GAPs

Members of the RHOGAP family possess a conserved 150-residue RHOGAP domain, which mediates the binding to the GTP-bound form of RHO proteins and accelerates their intrinsic GTP hydrolysis activity [26], thereby converting RHO GTPases to their inactive, GDP-bound form. GAPs therefore act as an inhibitor of RHO GTPases, and are often considered signal terminators. There are 66 members of the RHOGAP family in humans, which far outnumbers the RHO GTPases, indicating that several RHOGAPs can impart specific functions to individual RHO GTPase [17]. It is necessary to tightly control the activity of each RHOGAP to ensure an appropriate balance between the GDP- and GTP-bound states of RHO proteins [14]. In addition to activating GTP hydrolysis, GAPs may act as RHO GTPase effectors to mediate other downstream target functions [11].

#### GDIs

In mammals, there are three RHOGDI proteins, which bind to distinct RHO GTPases [14]. Among them, RHOGDI1 is the best-characterized and most abundant. It is extensively expressed and interacts with various RHO GTPases, including RHOA, RHOC, CDC42, RAC1, and RAC2 [27]. RHOGDI3 is usually expressed at low levels and is likely to interact with RHOB and RHOG [28]. RHOGDIs consist of a C-terminal domain, including a geranylgeranyl-binding pocket that is indispensable for extracting geranylgeranylated RHO GTPases from the membrane, as well as an N-terminal domain which interacts with the switch I and switch II domains of RHO GTPases, inhibiting exchange and hydrolysis to restrict GDP/GTP cycling [27, 29]. Thus, RHOGDI can form an inactive complex with RHO GTPases, and thereby sequester them to maintain inactive pools of RHO GTPases in the cytosol [14]. This complex also allows inactive RHO GTPases to quickly be translocated to any membrane in the cell to rapidly respond to specific signals [27]. In addition, they can serve as a chaperone to carry RHO GTPases between membranes, protecting the prenyl group of the RHO GTPase in the hydrophobic pocket. RHOGDI may therefore also contribute to the activation of RHO GTPases. Taken together, RHOGDIs prevent the inappropriate activation of RHO GTPases from and protect them from misfolding and degradation [30]. The classical model of the RHO GTPase cycle assumes that GDIs only extract with a GTPase when it has been inactivated by GAP [27]. However, recent studies indicate that RHOGDIs can not only promote the passive shuttling of inactive RHO GTPases (GDP-bound) in the cytoplasm, but also extract active RHO GTPases (GTP-bound) form membranes [31], which indicates that RHOGDIs effectively contribute to the spatiotemporal control of RHO GTPases.

Another aspect of the complex RHO regulation is the fact that GEFs and GAPs are multidomain proteins subject to complex regulation, which is modulated by posttranslational modifications, protein interactions, and binding of second messengers, which in turn regulate their localization, specificity, and activity [22]. In addition to regulation by GEFs, GAPs and GDIs, RHO GTPases are also controlled by post-translational modifications, including lipid modifications, ubiquitination, phosphorylation, and SUMOylation, which can significantly affect their function [16], especially in atypical RHO GTPases. Likewise, the expression of RHO GTPases can be regulated at the transcriptional or post-transcriptional level. For example, micro-RNAs (miRNAs) can regulate the posttranscriptional processing of RHO GTPase-encoding mRNAs [32]. These factors create a complex network of interactions that determine the precise spatiotemporal activation of RHO GTPases, and thereby determine their final effects on the cell.

### Cellular functions of RHO GTPases

**Fig. 4:**
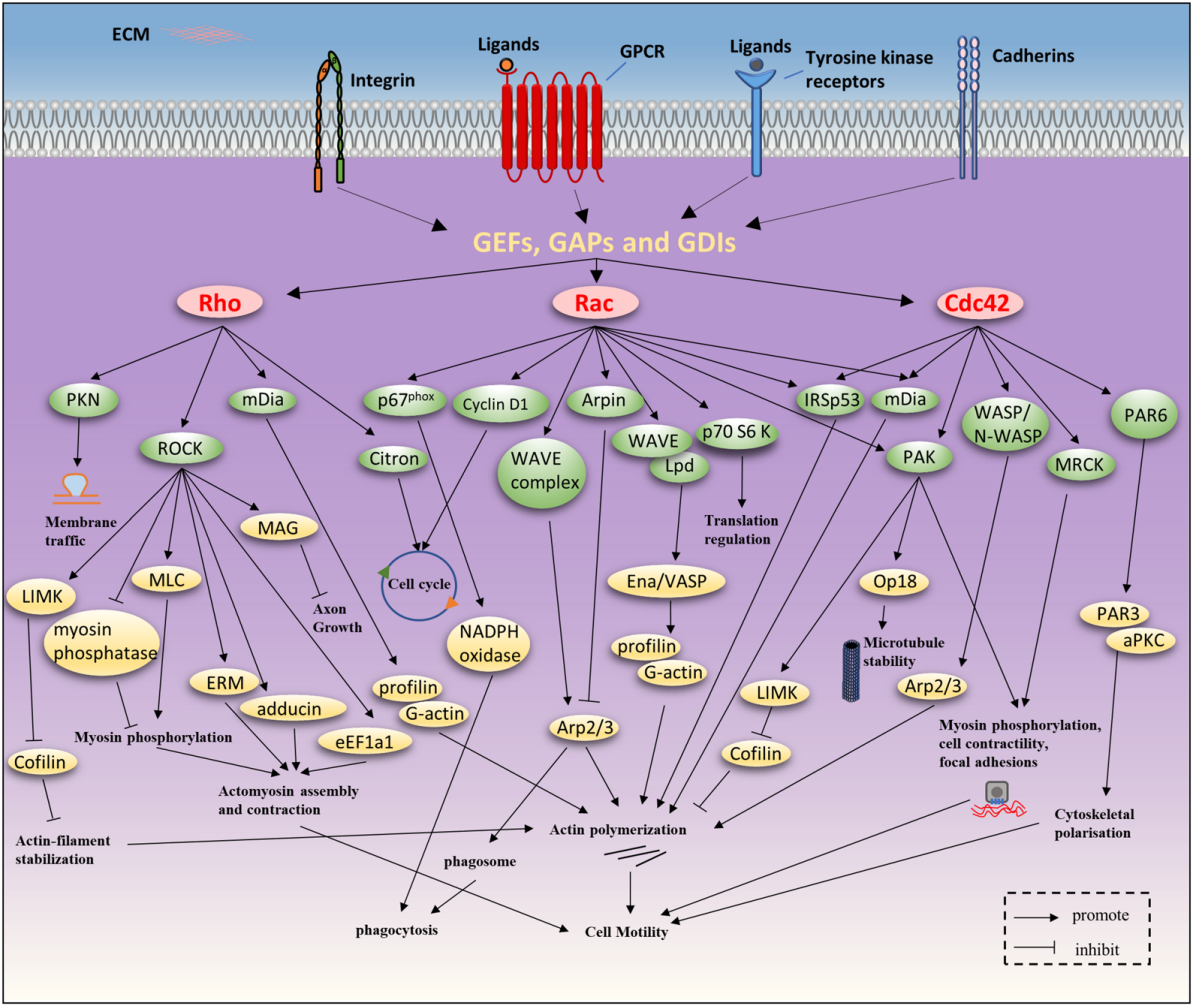
RHO GTPase function. Under stimulation by various upstream signals, including integrin, GPCRs and tyrosine kinase receptors, RHO GEFs are activated. Subsequently, activated RHOA, RAC1 and CDC42 bind to and specifically activate their downstream effectors, including protein kinases (e.g., PKN, ROCK, and Citron) and scaffolding proteins (e.g., WASP, IRSp53 and mDia). These target proteins activate distinct signaling pathways with multiple roles in cell motility, cell cycle, phagocytosis and membrane trafficking

Under stimulation by diverse upstream signals, RHO GTPases play various roles in cell motility, cell cycle, phagocytosis, membrane trafficking and other aspects (Fig. 4).

#### Cell motility

**Fig. 5:**
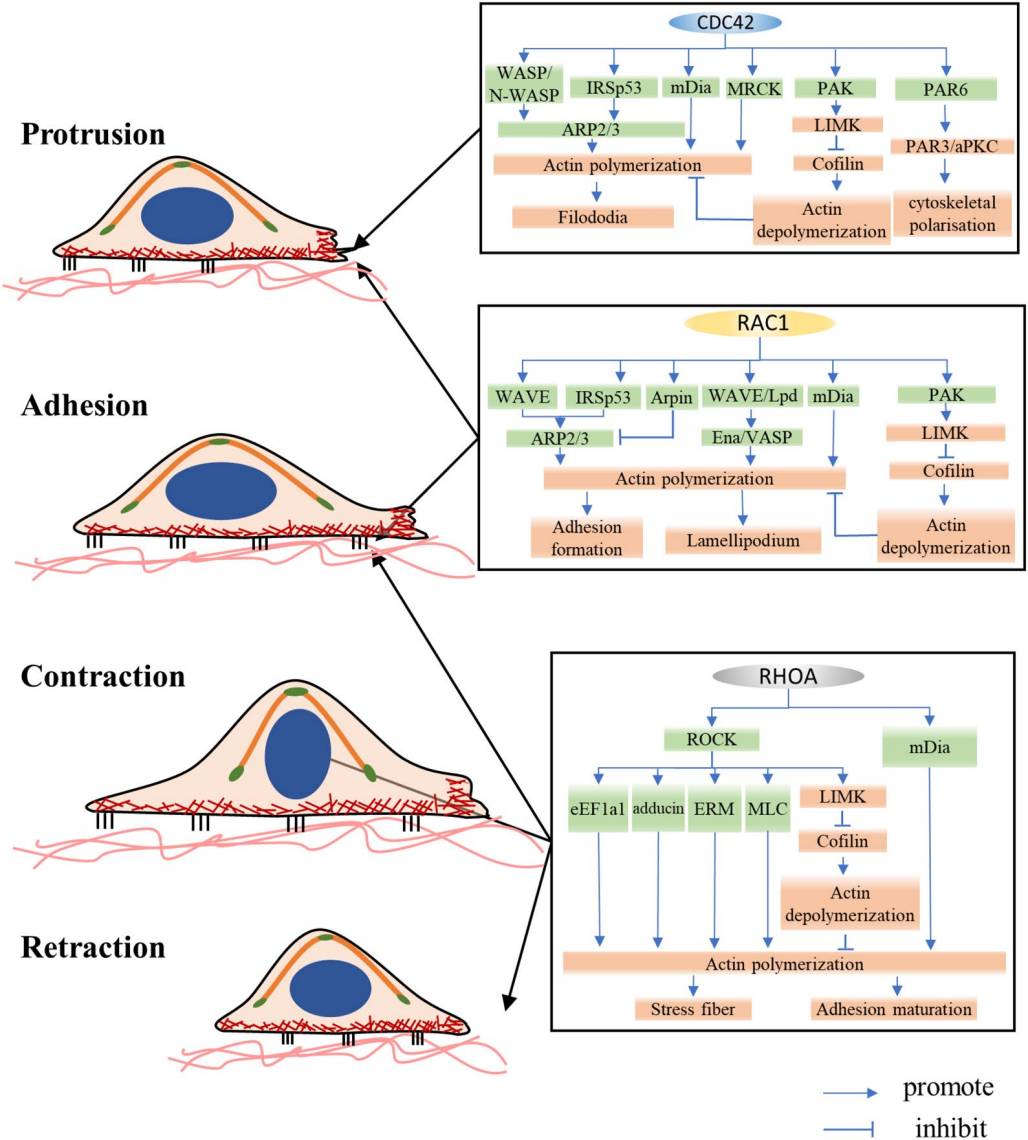
RHO GTPase-driven cell migration modes. As a response to various signals, migrating cells enter the cell motility cycle. At the leading edge, RAC1 induces the formation of actin-rich lamellipodia. CDC42 determines the formation of filopodia and the direction of motion. New protrusions adhere to the ECM through the formation of focal complexes, which are controlled mainly by RAC1 and RHOA. Then the cell body contracts depending on the formation of stress fibers and the contraction of actin-myosin fibers, which is mediated by RHOA and ROCKs. Finally, the rear adhesions are dissolved, the cell tail retracts, and the cell moves forward

Cell motility is a multistep process (Fig. 5), including (1). Protrusion of the leading edge, (2). Local formation of new adhesions, (3). Cell body contraction, and (4). Detachment of the trailing edge [33].

The initial step of cell migration is the extension of cytoplasm, which is induced by actin polymerization at the leading edge and determines the movement direction. Membrane protrusion involves new actin polymerization and requires actin nucleators, such as Ena/VASP protein and the actin-related protein-2/3 (Arp2/3) complex. The structure of membrane protrusions can be divided into finger-like structures called filopodia and sheet-like structures named lamellipodia, which are respectively induced by CDC42 and RAC1. CDC42 is activated in response to various external stimuli such as chemo-attractants, integrins, or receptors for soluble ligands. By releasing intramolecular interactions, CDC42-GTP can relieve the autoinhibitory mechanism of Wiskott–Aldrich syndrome protein (WASP) and Neuronal-Wiskott–Aldrich syndrome protein (N-WASP) to activate them. When activating these effectors, CDC42 contributes to the indirect activation of the Arp2/3 complex [34] to initiate peripheral actin polymerization [6], leading to filopodia formation. CDC42 can also induce cytoskeletal polarization through the polarity protein partitioning defective-6 (PAR6) pathway with PAR3 and/or isoforms of atypical protein kinase C (aPKC) [35]. Furthermore, CDC42 also plays important roles in cytoskeleton regulation and cell motility through many targets including the formin-family protein mammalian diaphanous (mDia), IRSp53 and myotonic dystrophy-related CDC42-binding protein kinases (MRCK) α/β [19, 36–38]. RAC activates the WASP-family verprolin-homologous protein (WAVE) complex, in turn which activates Arp2/3 to stimulate the formation of a “dendritic” actin network together with WAVE, contributing to lamellipodium extension [39, 40]. IRSp53 is another RAC target that contributes to this process by binding to RAC and WAVE [41]. However, RAC can also control the termination of Arp2/3 activation through Arpin [42]. RAC possibly also activates mDia2, which nucleates unbranched actin filaments [19]. By directly regulating the interaction between Lamellipodin (Lpd) and the WAVE complex, RAC activates Ena/VASP, which regulates the length of actin filaments at the cell front by temporarily safeguarding actin filament ends against capping protein while also recruiting polymerization-competent profilin-bound G-actin [43]. The binding of active RAC/CDC42 disturbs the autoinhibitory conformation of PAK and then activates its catalytic domain through phosphorylation [36]. RAC/CDC42 phosphorylates LIM motif-containing protein kinase (LIMK) to activate it via PAK1, which in turn efficiently induces the phosphorylation and inactivation of cofilin, leading to decreased depolymerization of F-actin [44]. As mentioned above, many effectors of CDC42 are also downstream of RAC, and CDC42 is consequently considered a potential regulator that drives the activity of RAC-dependent lamellipodia [45]. Strikingly, RHOA was found to be activated at the leading edge to promote the polymerization of actin [45]. Consequently, the combined activity of RAC, CDC42 and RHOA induces the formation of a protrusion at the leading edge to facilitate cell migration. Next, RAC and RHOA induce the formation of focal adhesions [46], in which actin-myosin fibers are connected to some discrete points on the inner plasma membrane, where dynamic protein complexes that are conducive to cell adhesion to the extracellular matrix (ECM) are localized [47].

Subsequent cell contraction is mainly mediated by stress fibers, a contractile device consisting of bundles of F-actin and myosin II [47]. The formation of stress fibers is mainly induced by RHOA/ROCK, which targets various cytoskeletal regulatory proteins. These proteins include the myosin light chain (MLC), MLC phosphatase, and LIMK [6]. ROCK phosphorylates and activates LIMK, which can further phosphorylate cofilin to inactivate it and inhibit its actin-depolymerization activity, resulting in the stabilization of actin filaments [48]. In addition, ROCK promotes the phosphorylation of MLC by directly phosphorylating it or inactivating myosin phosphatase, which finally causes myosin II activation and actomyosin-driven contractility [49]. mDia, another significant effector of RHOA, can cooperate with ROCK to assemble actomyosin bundles [50, 51]. Furthermore, ezrin/radixin/moesin (ERM), eukaryotic elongation factor 1-α1 (eEF1a1), and adducin are downstream effectors of ROCK that engage in actin cytoskeleton assembly [52]. Thus, RHOA not only strengthens actin polymerization, but also reduces its depolymerization to drive cell body contraction. Finally, RHOA/ROCK mediates tail retraction at the rear of the cell [46].

An epithelial-mesenchymal transition (EMT) is a biological process, which allows a polarized epithelial cell to assume a mesenchymal cell phenotype, playing important roles in embryo implantation, embryogenesis, organ development, tissue regeneration, organ fibrosis well as cancer progression and metastasis [53]. There are two migration fashions of invasive cells—a mesenchymal or an amoeboid mode of migration [54], and the core of the former is RAC and the latter is RHOA. RAC1 is closely related to the mesenchymal migration mode of invasive cells and is characterized by the elongated morphology of cells and a leading edge with active membrane ruffles [54]. Furthermore, the activity of RAC1 is central to modulating the switch between amoeboid and mesenchymal migration fashion. RAC1 activates WAVE2 to promote mesenchymal movement by actin assembly and to inhibit actomyosin contractility and amoeboid movement by suppressing ROCK activity [55]. In contrast, during amoeboid movement, ROCK inhibits RAC1 via stimulating ArhGAP22, a RAC GAP [55]. Amoeboid migration is characterized by round cells which have weak adhesion with surrounding matrices [56]. Increased RHOA induces amoeboid motility via stimulating membrane blebbing through ROCK-dependent phosphorylation of myosin II and consequent actomyosin contractility [56]. This migration approach displays high levels of actomyosin contractility and can squeeze through the matrix via deforming the cell body, in a proteolysis-independent way [57]. Furthermore, RHOA/ROCK can also promote EMT by upregulating EMT-related genes via AP-1 [58].

#### Cell cycle

The cell cycle includes four phases, termed G1, S, G2, and M. In order to avoid abnormal proliferation or apoptosis caused by abnormal passage through the cell cycle, there are many checkpoints that can arrest the cell cycle through proteins such as cyclins and cyclin-dependent kinases [59]. RHOA/ROCK induces the phosphorylation of glycogen synthase kinase-3β (GSK-3β) and accumulation of β-catenin, which contributes to increased expression of c-Myc and cyclin D1, leading to cell proliferation and migration [60]. RHOA is also involved in the cell cycle by Citron to regulate cytokinesis [61]. RAC1 is important for cellular transformation through the regulation of antiapoptotic signals and cell cycle machinery. RAC1 promotes the progression of the G1 phase by promoting the biosynthesis of cyclin D1 [62]. RAC1 can in turn also be activated by cyclin-dependent kinase 1 (CDK1) to promote mitosis through PAK [63]. In addition, RAC1 influences transformation by regulating nuclear factor-κB (NF-kB) [64]. By activating NF-κB in the nucleus, RAC1 leads to an inflammatory response that induces the nuclear translocation of nuclear factor erythroid 2-related factor 2 (NRF2) and increases its activity, which can block RAC1-dependent NF-κB activation through the NRF2/ARE pathway [65]. Thus, RAC1 has a unique nuclear function. In addition, RAC1 was also reported to control cell proliferation and transcription through p70 ribosomal S6 kinase (p70S6k) [66, 67].

#### Phagocytosis and membrane trafficking

Phagocytosis plays multiple roles in the organism, including tissue homeostasis, remodeling, and immune defense. RHO GTPases, especially RAC1, are involved in phagocytosis. RAC1/2 modulate Arp2/3 recruitment and actin polymerization at the phagosome to regulate phagocytosis mediated by integrins and Fc-gamma receptors (FcγRs) [68]. In phagocytic cells, RAC1 was found to regulate the activity of NADPH oxidase through p67 to promote phagocytosis [69]. Recent studies demonstrated that RAC1 and RHOA can induce phagocytosis of apoptotic bodies in hepatic stellate cells (HSCs) [70]. Moreover, RHO GTPases participate in membrane trafficking. RHOA is involved in endosomal trafficking by protein kinase N (PKN) [71]. Furthermore, CDC42/RAC1 is also involved in microtubule dynamics by inhibiting microtubule plus end disassembly through phosphorylation of Op18/stathmin to inactive it via PAK [6].

#### Other functions

RHO GTPases also have many other features. For instance, RHOU activates PAK1 and JNK1 to induce filopodia and regulate intercellular tight junctions [72]. RND proteins have been demonstrated to modulate the organization of the actin cytoskeleton in some tissues, participate in neurite extension, and regulate contractility of smooth muscles [21]. RHO GTPases play essential roles in the nervous system, for example, RHOA inhibits axon outgrowth by mediating the effects of myelin-associated axon growth inhibitors such as myelin-associated glycoprotein (MAG) through ROCK [73]. There are also findings implicating RHO GTPases in the regulation of metabolism. RAC1, which enhances the translocation of glucose transporter 4 (GLUT4) through a RalA-dependent downstream pathway, was shown to have the ability to regulate glucose uptake [74].

However, there are still many issues in the research of RHO GTPases, especially concerning atypical RHO GTPases such as RHOBTB, RND, RHOH, RHOV and RHOU. Further questions remain surrounding their regulatory mechanisms, the nature of their downstream signals, and also their functions in the cell and whole organism.

## RHO GTPase family in HCC

Hepatocellular carcinoma (HCC) is the fifth most common cancer in the world, as well as the third leading cause of cancer-related deaths [75, 76]. Moreover, the annual incidence of HCC is expected to surpass 1 million by 2025 [77]. HCC commonly occurs in the context of chronic liver disease due to risk factors such as hepatitis B or C, alcohol abuse or nonalcoholic hepatic steatosis and diabetes [78]. While novel diagnostic approaches and therapeutic strategies have led to substantial improvement, the long-term prognosis of patients with HCC remains unsatisfactory, with median 5 year survival of 18% [78], and metastasis as the main potential reason for high mortality [79]. Moreover, the median survival of patients with advanced HCC is only 6–8 months, and the main molecularly targeted drug, sorafenib, can only extend the median overall survival to 10.7–14.7 months [80, 81].

**Fig. 6:**
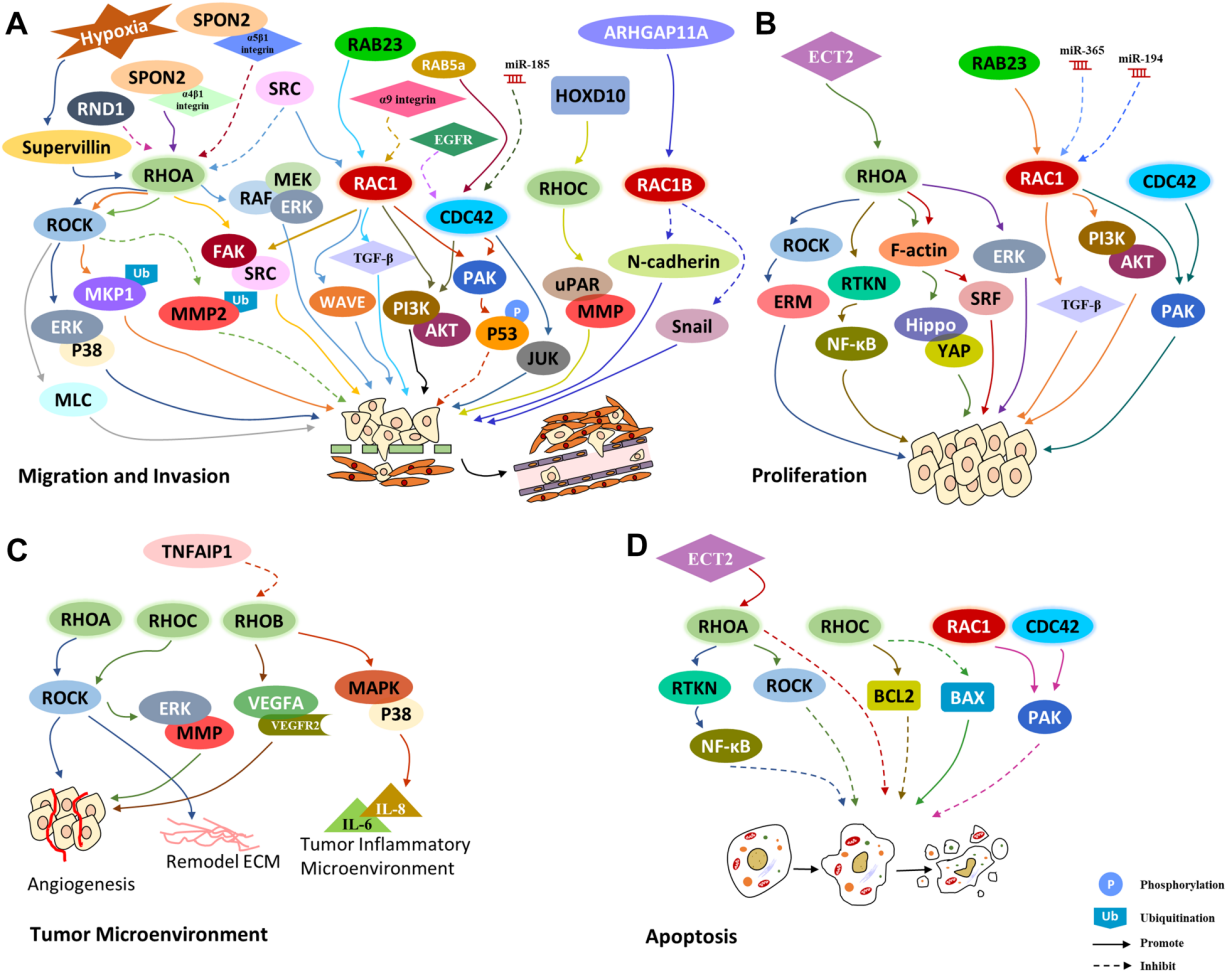
RHO GTPase in HCC.RHO GTPases promote HCC progression via various approaches and play an essential role in HCC **A** migration and invasion, **B** proliferation, **C** tumor microenvironment and **D** apoptosis

Strictly controlled cell migration is indispensable for the development of multicellular organisms, and its deregulation is a hallmark of metastatic cancer [82]. Recent studies have shown frequent dysregulation of RHO GTPases in a variety of human cancers, which is mainly caused by the dysregulation of their upstream regulators, GEFs, GAPs and GDIs. RHO GTPases have a well-recognized role in the acquisition of malignant features by cancer cells, and contribute to the modulation of aggressive biological behaviors of tumor cells by affecting the cytoskeleton [12]. Given that RHO GTPases participate in various cellular functions, there is no doubt that they are connected with almost every stage of cancer development and progression, such as the dysregulation of cell proliferation, angiogenesis, resistance to apoptosis, tissue invasion, and metastasis [14]. Therefore, exploring the roles of the RHO GTPase family in HCC initiation and progression may contribute to finding a cure for this aggressive malignancy (Fig. 6).

### RHO GTPases in HCC cell migration and invasion

The ability of cancer cells to spread to other parts of the body via metastasis is an important feature of cancer and a key factor determining the prognosis [83]. It is well known that RHO GTPases modulate cell motility and hence play an essential role in the migration, invasion, and metastasis of cancer cells [36, 84].

Many studies show that upregulation of RHOA expression promotes migration and invasion of HCC cells. For example, RHOA/ROCK can be activated by hypoxia-induced upregulation of supervillin to activate the extracellular regulated protein kinase (ERK)/p38 pathway, leading to the promotion of HCC cell migration and invasion [85]. Recent studies found that RHOA is frequently overexpressed in HCC, which is strongly correlated with satellite lesions, venous invasion and advanced TNM stage [86, 87]. Additionally, RHOA/ROCK2 was reported to promote the ubiquitination and degradation of dual-specificity phosphatase-1 (DUSP1/MKP1) to downregulate the protein expression of MKP1, resulting in the promotion of HCC invasion [88]. RHOA can also block the ubiquitination and degradation of MMP2 via ROCK2 to facilitate invasion of HCC [89]. Another study demonstrated that RHOA promotes HCC cell migration and invasion by activating ROCK1/MLC signaling, and facilitates HCC metastasis by enhancing the activity of the FAK/SRC pathway [90]. Moreover, RHOA activity can also be inhibited by Snail to recruit SRC-phosphorylated p190 RHOGAP, thereby promoting collective migration and the formation of circulating tumor cell clusters [91]. When activated by transcription factor HOXD10, RHOC promotes HCC metastasis via the urokinase-type plasminogen activator receptor (uPAR)/MMP pathway [92]. Another study demonstrated that RHOA can be activated by matricellular protein SPON2-α4β1 integrin signaling, and inactivated by SPON2-α5β1 integrin signaling or integrin α9 to control HCC cell migration [93, 94].

In contrast to primary HCC cell lines, an elevated active RAC1 level was found in a metastatic HCC cell line [95]. RAC1-WAVE2 signaling can be activated by SRC, which inhibits RHOA-ROCK signaling at the same time, leading to mesenchymal-type movement of HCC cells [96]. Following activation by RAB23, RAC1 increases the expression of TGF-β, resulting in the promotion of EMT and migration of HCC cells [97]. The activity of RAC1 can be suppressed by integrin α9 to decrease the phosphorylation of FAK and SRC, thereby inhibiting the migration and invasion of HCC cells [94]. In addition, elevated expression of RAC1B, a RAC splice variant with more repaid GDP/GTP exchange, was found in many cancers[98–100], including liver cancer [101]. The expression of RAC1B can be enhanced by ARHGAP11A to promote invasion, migration and EMT of HCC cells by decreasing N-cadherin and Snail expression while increasing E-cadherin expression [101].

However, CDC42 has contradictory effects in HCC. Although CDC42 was found to be upregulated in a variety of tumors [16], the loss of CDC42 in the liver leads to tumorigenesis and progressive development of HCC [102], suggesting a potential tumor suppressor role of CDC42 [102]. Strikingly, CDC42 was found to enhance the ability of HCC cells to invade surrounding tissues by inducing filopodia formation [103]. The expression of CDC42 in HCC can be increased by decreasing the expression of epithelial growth factor receptor (EGFR) to induce Myosin II activation, thus promoting HCC migration and invasion [104]. The transcriptional activity of the CDC42 promoter can be increased by RAB5a to upregulate CDC42 expression and thereby facilitate HCC progression [105]. Conversely, miR-185 was found to suppress this process by directly downregulating CDC42 expression [106]. In addition, RAC1 and CDC42 are correlated with cancer metastasis, upregulating the PI3K signaling pathway to promote the migration and invasion of various types of cancer, including HCC [107]. Furthermore, RAC1/CDC42 induces the phosphorylation of Ser215 of p53 by PAK4, further eliminating the inhibitory effect of p53 on the invasion and migration of HCC cells [108]. RAC1/CDC42 also promotes HCC EMT and metastasis via PAK1 [109, 110], which induces cancer metastasis via the phosphorylation of paxillin and activation of JNK [111].

Several other atypical RHO GTPases also play important roles in HCC metastasis, such as RHOF, which interacts with AMP-activated protein kinase (AMPK) and enhances its phosphorylation. This in turn increases RAB3d expression, amplifying the Warburg effect to promote the migration and invasion of HCC cells [112]. It was also reported that RND1 is downregulated in HCC, enhancing the activity of RHOA and leading to EMT-mediated migration and metastasis of HCC cells via the RAF/MEK/ERK signaling pathway [113].

Consequently, RHO GTPases are considered a key factor in regulating the migration, invasion, EMT and metastasis of HCC cells.

### RHO GTPase family in HCC cell proliferation

In the early stages of HCC development, uncontrolled cell proliferation is critical, and RHO GTPase family is involved in the dysregulation of proliferation. For example, RHOA activates the ERM pathway and EMT via ROCK1 to facilitate HCC cell proliferation [114]. Furthermore, RHOA/ Rhotekin (RTKN) promotes HCC cell proliferation by activating NF-κB signaling [115]. RHOA can also promote HCC cell proliferation and cell cycle progression by upregulating cell cycle-associated proteins, CDK1 levels and proliferating cell nuclear antigen through RTKN2 [116]. Another study demonstrated that RHOA can also be activated by epithelial cell transforming sequence 2 (ECT2) to facilitate the proliferation of HCC cells via the RHOA/F-actin/Hippo-YAP signaling axis [117]. Similarly, RHOA/actin promotes HCC cell proliferation via the transcriptional regulator serum response factor (SRF) [118]. Other studies showed that RHOA facilitate HCC cell proliferation not only via ROCK2 [119], but also ERK [120]. Of note, RHOC can promote cell cycle progression by upregulating cyclin D1 and CDK4 as well as downregulating cyclin-dependent kinase inhibitors, p16 and p21, thus promoting HCC cell proliferation [121]. RAC1 can also be activated by RAB23 to increase the expression of TGF-β or activate PI3K/AKT signaling, leading to the growth and proliferation of HCC cells [97, 122]. Conversely, RAC1 can be inhibited by miR-365 and miR-194, leading to the suppression of HCC dedifferentiation and cancer stem cell proliferation [123, 124]. In addition, RAC and CDC42 are able to inhibit cell cycle arrest in HCC via PAK5 and thereby facilitate tumor growth cells [125]. Strikingly, RHOE enables HCC cell to bypass senescence and promotes their proliferation [126].

Thus, the crucial roles of RHO GTPases in cell proliferation are becoming increasingly clear, providing a theoretical basis for a deeper understanding of HCC pathogenesis.

### RHO GTPases in HCC microenvironment

RHO GTPase family has important regulatory roles in inflammation and angiogenesis in the tumor microenvironment of HCC. Tumor capillary endothelial cells (ECs) show abnormally high levels of RHOA, resulting in aberrant mechanosensing and excessive angiogenesis [127]. RHOA/ROCK can also remodel the ECM in the tumor microenvironment to facilitate HCC cell invasion [128, 129]. RHOC/ROCK2 promotes vasculogenic mimicry (VM) in HCC through ERK/MMPs, which significantly improves the tumor blood supply [130]. Furthermore, knockdown of RHOC in HCC cells reduced VEGF expression as well as the migration and organization of ECs to decrease HCC-induced angiogenesis [131]. In addition, RHOB was also found to promote angiogenesis by enhancing VEGFA-VEGFR2 signaling to contribute to HCC malignancy [132]. The degradation of RHOB is blocked by TNFAIP1 downregulation, thereby activating the p38/JNK MAPK pathway to induce the expression of IL-6 and IL-8 in TNF-α-stimulated HCC cells [133]. These pro-inflammatory cytokines can modulate the tumor inflammatory microenvironment to facilitate cancer development and progression.

Conversely, the expression and activity of RHO GTPases can also be modulated by the tumor microenvironment. For instance, a recent study found that the expression of RHOA/ROCK and RAC1/PAK can be increased by hypoxia, inducing VM through the stabilization of HIF-1α and p-Vimentin-activated EMT, which ultimately promotes the invasion and metastasis of HCC [134].

### RHO GTPase in hcc cell apoptosis

Escape from apoptosis is a hallmark of cancer [135], and the RHO GTPase family is also involved in the process of apoptosis. For example, RHOA inhibits HCC cell apoptosis via RTKN [116], which can activate NF-κB signaling [115]. Moreover, RHOA can also block apoptosis of HCC cells through ROCK2 [119, 136]. RHOA also inhibits HCC cell apoptosis when activated by ECT2 [137]. Similar to RHOA, RHOC plays an antiapoptotic role in HCC by upregulating the antiapoptotic gene BCL2 [138], and downregulating the proapoptotic gene BAX [121]. Another study unambiguously showed that CDC42/RAC1 mediates cisplatin resistance by inhibiting cell cycle arrest and apoptosis in HCC cells through PAK5 [125]. However, the exact mechanism requires further studies.

## Treatment strategies

While novel diagnostic approaches and therapeutic strategies have substantially improved, the cure rates and long-term survival of patients with HCC remain unsatisfactory [76]. Hence, there is an urgent need to explore the molecular mechanisms underlying tumorigenesis, metastasis and chemoresistance in HCC to identify new therapeutic targets.

**Fig. 7:**
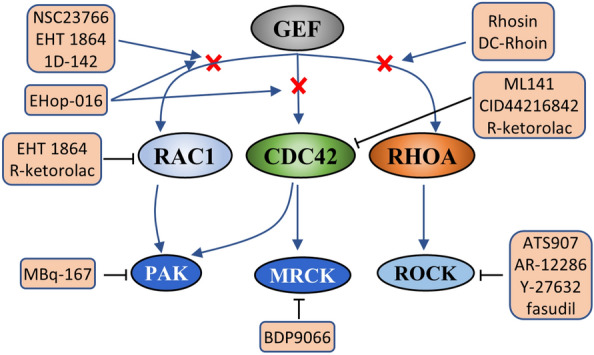
Targeting the RHO GTPase signaling. There are many approaches to target RHO GTPase signaling, including disrupting the interactions between RHO and GEF, inhibiting RHO proteins directly and inhibiting the effect of RHO effectors

As mentioned above, the dysregulation of RHO GTPases is involved in the various aspects of malignancy, which makes RHO GTPase family an appealing target for cancer therapy [12, 13]. However, because of the sub-nanomolar binding affinity of RHO GTPases for their substrates GDP or GTP, as well as the high concentration of GTP in the cells [139], and the lack of any suitable binding sites in RHO GTPases, they are generally presumed to be undruggable clinical targets. Therefore, drug development for targeting RHO family proteins is limited. So far, the major pharmacological interventions targeting RHO GTPases are the lipid modification on their carboxy-terminal region, the interface of RHO with RHOGEF [139], and downstream effectors [13] (Fig. 7, Table. 1). Nevertheless, because of many problems such as high toxicity, low selectivity and insufficient efficacy of existing inhibitors, there are no clinically efficacious drugs targeting RHO GTPases for cancer treatment available [140].

**Table 1:** Inhibitors for RHO GTPases

Target	Name of compound	Mechanism	Effect	Ref.
RHO GTPases				
RAC	EHT 1864	Lock in inactive state	Blocks migration and invasion of cancer cell	[141]
RAC/CDC42	R-ketorolac	Targets RAC1 and CDC42	Blocks migration and invasion of cancer cell	[142]
CDC42	ML141	Block nucleotide binding	Blocks migration and invasion of cancer cell	[143]
RHO/RAC/CDC42	statins	Targeting HMG CoA reductase pathway to diminishing lipid modifications needed by Rho GTPases	Blocks proliferation and invasion of cancer cell	[146, 147]
Interaction of RHO and effectors				
ROCK	ATS907	Compete with ATP	In trial for glaucoma and ocular hypertension	[148]
	AR-12286	Compete with ATP	In trial for glaucoma and ocular hypertension	[148]
	Y-27632	Compete with ATP	Blocks proliferation, migration and invasion of cancer cell	[151]
	fasudil	Compete with ATP	Blocks proliferation, migration and invasion of cancer cell	[152, 153]
PAK	MBQ-167	Blocks PAK phosphorylation	Blocks growth, migration and EMT of cancer cell	[155]
MRCK	BDP9066	Compete with ATP	Blocks proliferation and invasion of cancer cell	[156]
Interaction of RHO and RHOGEF				
RAC	NSC23766	Blocks interaction of RAC1 with TIAM1 and Trio	Blocks migration and invasion of cancer cell	[157–159]
	1D-142	Block GEF binding	Blocks growth and invasion of cancer cell	[161]
RAC/CDC42	EHop-016	Derivative of NSC23766	Blocks migration and invasion of cancer cell	[160]
RHOA	Rhosin	Block GEF binding	Blocks migration and invasion of cancer cell	[162]
	DC-Rhoin	Binds to the surface of RHOA by Cys107	Blocks migration and invasion of cancer cell	[140]

### Inhibitors targeting the RHO GTPase

There are two approaches to inhibiting RHO GTPase: inhibiting RHO-nucleotide interactions or regulating the localization of RHO. For instance, EHT 1864, a nucleotide-binding inhibitor of RAC, which suppresses both guanine nucleotide association and Rac1-Tiam1 complex formation, keeping Rac in an inactive state [141]. R-ketorolac, a specific inhibitor of RAC and CDC42, can inhibit cancer cell migration and invasion in vivo [142]. Otherwise, the specific CDC42 inhibitor ML141 (CID2950007) and its analog CID44216842 can inhibit GTP binding to CDC42 to block CDC42-driven cancer cell migration [143]. Because of the absence of stable binding pockets on the surface of RHOA, the generation of RHOA-specific inhibitors presents several challenges [144]. Furthermore, membrane association is a prerequisite of RHO GTPase activation, whereas their ability to anchor at the membranes depends on the presence of the prenyl group. Splice variants of SmgGDS are major regulators of the prenylation of RHO family members [145]. However, statins can increase SmgGDS expression via targeting HMG CoA reductase pathway and thus diminishing lipid modifications needed by Rho GTPases to suppress their activation [146, 147].

### Inhibitors targeting the interaction of RHO and their effectors

A more common strategy to suppress the role of RHO GTPases in facilitating malignancy is targeting its downstream effector proteins. Most kinase inhibitors target the kinase ATP binding site in the active state of kinase, reversibly competing with ATP [148]. As mentioned above, the RHOA effector ROCK plays a promoting role in various stages of cancer. Even though considerable efforts have been made to develop ROCK inhibitors, most are still at the preclinical evaluation stage. For example, studies of ATS907 and AR-12286 for glaucoma were discontinued due to their adverse effects [148]. Y-27632 consistently suppresses RHOA-induced, ROCK-mediated formation of focal adhesions and stress fibers [149, 150], and inhibits intrahepatic metastasis of human HCC [151]. In Japan and China, the ROCK inhibitor fasudil is approved for the acute clinical treatment of cerebral vasospasm [152] and shows therapeutic potential in HCC [153]. However, its pharmacokinetic characteristics make it unsuitable for use in chemotherapy [154]. The high similarity of the homologous ATP-binding regions of ROCK and several other protein kinases, such as PKA and PKC, restricts the development of highly selective ROCK inhibitors [52, 148]. Taken together, ROCK inhibitors may have significant potential for treating cancer and other diseases, with clinical trials for human cancers currently under way [148]. Furthermore, the RAC1/CDC42 inhibitor MBQ-167 may be a promising anticancer drug that specifically suppresses downstream PAK signaling and STAT3 activity to inhibit the growth, migration and EMT of cancer cells [155]. Another study found that BDP9066 is a selective small-molecule inhibitor of the Cdc42-binding MRCK kinases [156]. Although rationally designed small molecule inhibitors have shown promising preclinical results, there are no clinically effective drugs approved for cancer treatment, and there are also no related clinical trials at present.

### Inhibitors targeting the interaction of RHO and RHOGEF

Another approach to design inhibitors of the RHO GTPase family is to target the interaction with their upstream regulators. NSC23766 can impair the interaction of RAC1 with TIAM1 and Trio GEFs, but cannot disturb the activation of RHOA and CDC42 [157]. Therefore, this inhibitor can suppress the CAMSAP2-dependent RAC1/JNK pathway, or the cysteine-rich domains-1-RAC1 pathway to inhibit the invasion and migration of human HCC [158, 159]. However, the potency of this compound is relatively low, limiting its further development as a clinical candidate. EHop-016 is another RAC1 inhibitor that binds more tightly to the effector domain, moreover, it is a derivative of NSC23766 but is 100 times more potent than NSC23766 [160]. However, EHop-016 is no longer specific to RAC1 at higher concentrations but also suppresses CDC42 activity without influencing RHOA. The specific RAC1 inhibitor 1D-142 can modulate the RAC1-related transcriptional programme in HCC via regulating the interaction of RAC and RACGEF to significantly inhibit tumor growth and intrahepatic metastasis [161].These inhibitors can be further developed as pharmacological inhibitors of RAC in metastatic cancer cells. Rhosin, the first developed RHOA-specific inhibitor, impedes the docking between RHOA and various GEFs to suppress cancer progression [162]. DC-Rhoin, a novel inhibitor of RHO GTPase, can covalently modify RHO protein at Cys107 to disrupt the interaction of RHO with GEF and GDI [140]. Because of the inactivation of RHO GTPase by RHOGAP [163], enhancing RHOGAP activity is a promising treatment strategy. However, several RHOGAPs overexpressed in HCC exert a negative effect on HCC progression, therefore confounding the development of RHOGAP activators as anticancer agents. Preventing the release of RHO GTPases from RHOGDIs may also be a potential inhibitory strategy.

Although inhibitors of RHO GTPase family have not been used clinically for cancer treatment to date, rational targeting to the RHO GTPase family still carries significant potential in the discovery of new anticancer drugs, particularly for future combinatorial therapies. We should further evaluate the benefits and risks of the inhibitors as well as develop inhibitors with higher potency and less toxicity.

## Conclusions

There is accumulating evidence for the crucial roles of the RHO GTPase family in the development of HCC. RHO GTPases directly control the movement of cancer cell, promoting migration, invasion and metastasis, ultimately leading to the EMT and a switch of cancer cell migration between mesenchymal and amoeboid modes. In addition, the RHO GTPase regulators GEFs, GAPs and GDIs are also usually dysregulated in HCC. Therefore, this subfamily is considered a promising therapeutic target for HCC. However, the development of inhibitors for RHO GTPases is limited due to several issues such as their intrinsic structure, which lacks stable binding pockets. We need to further clarify the regulatory mechanism of the RHO GTPase family to open up novel directions for the design of additional therapeutic interventions and pay more attention to the design of inhibitors targeting RHO effectors and regulators. Furthermore, the majority of studies on the roles of RHO GTPase and its regulators in cancer progression have been performed in vitro. To understand whether they also contribute to the migration and invasion of cancer cells in vivo, future studies should establish pre-clinical in vivo models. Finally, we mainly focus on RHOA, RAC1 and CDC42, with few studies investigating the other members. Therefore, further studies are needed to understand the roles of these less-characterized RHO GTPases in the development of HCC.

## Data Availability

Not applicable.

## Abbreviations

GDIs: Guanine nucleotide dissociation inhibitors
GEFs: Guanine nucleotide exchange factors
GAPs: GTPase-activating proteins
DOCK: Dedicator of cytokinesis
DH: DBL-homology
miRNAs: Micro-RNAs
WASP: Wiskott–Aldrich syndrome protein
N-WASP: Neuronal-Wiskott–Aldrich syndrome protein
Arp2/3: Actin-related protein-2/3
PAR6: Partitioning defective-6
aPKC: Atypical protein kinase C
mDia: Mammalian diaphanous
MRCK: Myotonic dystrophy-related CDC42-binding protein kinases
WAVE: WASP-family verprolin-homologous protein
Lpd: Lamellipodin
ECM: Extracellular matrix
MLC: Myosin light chain
LIMK: LIM motif-containing protein kinase
ERM: Ezrin/radixin/moesin
eEF1a1: Eukaryotic elongation factor 1-α1
GSK-3β: Glycogen synthase kinase-3β
CDK1: Cyclin-dependent kinase 1
NF-kB: Nuclear factor-κB
NRF2: Nuclear factor erythroid 2-related factor 2
p70S6k: P70 ribosomal S6 kinase
FcγRs: Fc-gamma receptors
HSCs: Hepatic stellate cells
PKN: Protein kinase N
MAG: Myelin-associated glycoprotein
RTKN: Rhotekin
GLUT4: Glucose transporter 4
HCC: Hepatocellular carcinoma
ERK: Extracellular regulated protein kinase
DUSP1: Dual-specificity phosphatase-1
uPAR: Urokinase-type plasminogen activator receptor
EGFR: Epithelial growth factor receptor
AMPK: AMP-activated protein kinase
ECT2: Epithelial cell transforming sequence 2
SRF: Serum response factor
ECs: Endothelial cells
VM: Vasculogenic mimicry
MDR: Multidrug resistance
YAP: YES-associated protein
mTOR: Mammalian target of rapamycin
EMT: Epithelial-mesenchymal transition
SSO: Splice-switching oligonucleotides
